# Piphillin: Improved Prediction of Metagenomic Content by Direct Inference from Human Microbiomes

**DOI:** 10.1371/journal.pone.0166104

**Published:** 2016-11-07

**Authors:** Shoko Iwai, Thomas Weinmaier, Brian L. Schmidt, Donna G. Albertson, Neil J. Poloso, Karim Dabbagh, Todd Z. DeSantis

**Affiliations:** 1 Informatics Department, Second Genome Inc., South San Francisco, California, United States of America; 2 Bluestone Center for Clinical Research and the Department of Oral and Maxillofacial Surgery, New York University College of Dentistry, New York, New York, United States of America; 3 Helen Diller Family Comprehensive Cancer Center, University of California San Francisco, San Francisco, California, United States of America; 4 Research and External Scientific Innovation Department, Allergan PLC, Irvine, California, United States of America; University of Oklahoma, UNITED STATES

## Abstract

Functional analysis of a clinical microbiome facilitates the elucidation of mechanisms by which microbiome perturbation can cause a phenotypic change in the patient. The direct approach for the analysis of the functional capacity of the microbiome is via shotgun metagenomics. An inexpensive method to estimate the functional capacity of a microbial community is through collecting 16S rRNA gene profiles then indirectly inferring the abundance of functional genes. This inference approach has been implemented in the PICRUSt and Tax4Fun software tools. However, those tools have important limitations since they rely on outdated functional databases and uncertain phylogenetic trees and require very specific data pre-processing protocols. Here we introduce Piphillin, a straightforward algorithm independent of any proposed phylogenetic tree, leveraging contemporary functional databases and not obliged to any singular data pre-processing protocol. When all three inference tools were evaluated against actual shotgun metagenomics, Piphillin was superior in predicting gene composition in human clinical samples compared to both PICRUSt and Tax4Fun (p<0.01 and p<0.001, respectively) and Piphillin’s ability to predict disease associations with specific gene orthologs exhibited a 15% increase in balanced accuracy compared to PICRUSt. From laboratory animal samples, no performance advantage was observed for any one of the tools over the others and for environmental samples all produced unsatisfactory predictions. Our results demonstrate that functional inference using the direct method implemented in Piphillin is preferable for clinical biospecimens. Piphillin is publicly available for academic use at http://secondgenome.com/Piphillin.

## Introduction

Clinically relevant microbiome studies have revealed the composition of bacterial communities in both health and disease based on ubiquitous use of 16S rRNA gene profiling technologies. Some studies have demonstrated specific strains contributing to certain mechanisms of host responses [1–3]; however, more frequently specific microbiome functions and their interactions with the host are unknown. The 16S rRNA gene sequencing methods measure the structure of the microbiome community and changes thereof. However, to obtain mechanistic information, functional analysis is necessary. Shotgun metagenomic sequencing may allow comprehensive detection and quantification of all functional genes in each biospecimen, allowing inference of possible function and generating testable hypotheses of mechanism. Nevertheless, a practical method of generating the sequencing depth required to access all minority species and assemble their novel genomes has not yet emerged. For example 1 microgram of DNA sheared into 500 bp fragments contains over 1 trillion molecules, a number far beyond what any NGS instrument can handle. Thus, inferring metagenomics content using 16S rRNA gene amplicon sequencing counts is appealing due to the increased likelihood of enumerating both dominant and minority species. Although inference of availability of specific enzymes can been drawn from 16S rRNA amplicons [4], it was Langille et al. who popularized a systematic procedure implemented in the tool PICRUSt [5]. The algorithm was the first to predict potential functions in the microbial communities using only sequenced 16S rRNA information. The algorithm requires: 1) a reference tree representing the phylogenetic relationships between all known bacteria and archaea positioned at the leaves and, 2) a conjecture of the ancestral genome contents of all hierarchical nodes. These steps are computationally time-consuming and can yield discordant interpretations when changing one of the necessary parameters to the chain of software tools required. No generally accepted recommendations have been devised for the selection of conserved genes, their alignment, the calculation method used when comparing their polymorphisms, nor the tree reconstruction software [6]. Collectively, these factors are barriers to frequent updates. In fact, the tree leveraged by PICRUSt is from 2013 [7].

Tax4Fun is another tool published recently that infers functional capabilities from 16S rRNA amplicon datasets. It requires an association matrix between the prokaryotic organisms in the KEGG database and the SILVA SSU Ref NR database as well as pre-computation of functional profiles for all prokaryotic genomes in KEGG. In addition, the input sequence data needs to be converted to a SILVA-based profile [8].

PICRUSt uses v3.5 of IMG with 2,590 genomes as its reference genome dataset, each with a corresponding 16S rRNA in the reference tree. As of October 2015, the IMG database has been updated to v4.540 with 4,292 bacterial genomes, a 66% increase over the current PICRUSt references not counting an additional 4,050 genomes in draft stage. The number of sequenced genomes will be continuously increasing due to the drop in genome sequencing costs. To take advantage of the expanding database of genomes for inferred metagenomics, a rapid and simple algorithm that leverages the up-to-date genome repository without depending on uncertain and time-consuming phylogenetic tree reconstruction is necessary.

Here, we report Piphillin, a new metagenomics inference tool that can easily work with any current genome database. The algorithm uses direct nearest-neighbor matching between 16S rRNA amplicons and genomes to predict the represented genomes. We examined identity cutoffs for determining the nearest-neighbor genome by using three different datasets that were sequenced by both shotgun metagenomics and 16S rRNA: paired human oral cancer biopsy samples [9], rat feces and hypersaline microbial mat samples [10,11]. We also compared Piphillin results to PICRUSt and Tax4Fun, a recently published tool dependent on the Silva ontology [12], regarding correlation to corresponding shotgun metagenomics, and sensitivity and specificity of differential abundance detection between experimental groups.

## Materials and Methods

### Ethics statement

Human oral biopsy samples were a part of a previous study [9] and approved by the Institutional Review Board of New York University College of Dentistry and all patients provided written informed consent. The rat feces study was approved by the Allergan Animal Use and Care Committee.

### Subjects

For human oral biopsy samples, a subset of patients enrolled for the previous study [9] was used in this study. Paired cancer and anatomically matched contralateral clinically normal regions of the oral cavity from 6 patients (total n = 12) were collected. Rat fecal samples were collected from male Sprague-Dawley rats on a normal chow diet (n = 7).

### DNA extraction, 16S rRNA PCR and sequencing

Sample DNA was extracted and quantified as described previously [9] for human oral biopsy samples and for rat fecal samples [13]. Amplification of the V4 region of the 16S rRNA was performed with the 515F and 806R bacterial universal primer set with attached Illumina sequencing adaptor (Illumina, San Diego, CA). Amplified products were sequenced using Illumina MiSeq instrument as described previously [13].

### Shotgun metagenomics sequencing

Preparation of the human oral biopsy DNA library was described previously [14]. The DNA libraries for the environment and rat fecal samples were prepared using the Illumina Nextera Kit (Illumina) following the manufacturer’s instructions and quality was assessed on the BioAnalyzer 2100 (Agilent, Santa Clara, CA). Paired-end libraries were sequenced with 100 bp read length from each end using Illumina HiSeq2000 (Illumina).

### Datasets

Paired cancer and anatomically matched contralateral clinically normal human oral biopsy samples are described in Schmidt et al. [9] [16S rRNA accession number, EMBL PRJEB4953; metagenomics accession numbers, SRR3586059—SRR3586070]. Hypersaline microbial mat data is described in [10,11] [16S rRNA accession numbers, JN427016–JN539989; metagenomics accession numbers, ABPP00000000—ABPY00000000]. All human feces data that have 16S rRNA and corresponding shotgun metagenome sequencing data were downloaded from NCBI according to the HMP website (http://hmpdacc.org/) [15].

### Reference database and 16S rRNA sequences

Gene copy numbers were retrieved from Kyoto Encyclopedia of Genes and Genomes (KEGG; http://www.genome.jp/kegg/) release 73 (January 2015) to create a gene feature table. From each genome sequence, 16S rRNA gene IDs were extracted using keyword “K01977” (16S ribosomal RNA) in xxx_genes.txt files (xxx represents the genome id). Corresponding fasta format 16S rRNA sequences were retrieved and filtered using a min-length of 1400 bp and a max-length of 1600 bp. The number of 16S rRNA sequences passing the length filter in each genome was recorded.

### 16S rRNA sequence processing

To pre-process 16S rRNA gene libraries for Piphillin functional inference, sequences were binned into OTUs, a representative sequence from each OTU was selected and the count of sequences in each OTU from each sample were tallied using a previously described method [13].

### Shotgun metagenomics analyses

All retrieved raw shotgun metagenomics sequences were processed using a single shotgun metagenomics pipeline. Sequences were adapter-trimmed using FastqMcf in ea-utils (http://code.google.com/p/ea-utils), quality-filtered using PrinseqLite [16] with parameters -min_len 35 -ns_max_n 2 -derep 1 -derep_min 4 -trim_qual_left 20 -trim_qual_right 20 -trim_qual_type sum, then host (human or rat) sequences were removed, if applicable, using Bowtie2 [17] with—very-sensitive-local option against hg19 (human host) or rn4 (rat host). Then, rRNA sequences were removed using SortMeRNA [18] and remaining sequences were searched against KEGG bacteria and archaea genome database by RAPSearch [19,20] with parameters -l 10 -w t. Search results were parsed using a custom script according to the following rule: if the query aligned to subject with ≥20 aa length and ≥80% similarity, then highest bit-score subject is considered as a gene hit. Metagenomics abundance table calculations were complicated by the observation that a single Illumina read can align equally well to multiple proteins representing more than one KEGG Ortholog (KO). Instead of omitting these reads, we included them and split the counts among the KOs.

### Inference of metagenomics by Piphillin

**Fig 1:**
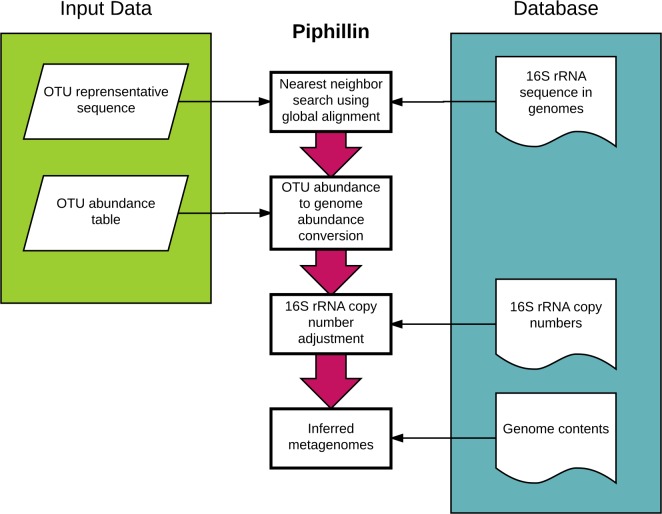
Piphillin algorithm. The representative sequence of each OTU in the sample is first searched against 16S rRNA sequences in the genome database to obtain inferred genome(s). Then the OTU abundance table is converted to a genome abundance table. The resulting table is normalized by the 16S rRNA copy number of each genome and a metagenome is inferred using the gene contents (copy number of each gene) of each genome in the database.

Piphillin was developed to utilize the most up-to-date genome databases to infer metagenomics content from16S rRNA sequenced samples (Fig 1). Piphillin can leverage any genome database with known gene contents for each genome. The web version of Piphillin (http://secondgenome.com/Piphillin) currently supports different releases of KEGG and BioCyc. In this study, we used KEGG as a reference database (results using BioCyc [21] as a reference database can be found in S1 Text). Gene copy numbers within each genome were retrieved, summarized by KO and formatted for the database using a custom script. In the Piphillin algorithm, genome contents were predicted for each OTU. It has been observed that high accuracy in predicting metagenomic content can be achieved by directly matching OTUs to the nearest sequenced genome without reliance on phylogenetic trees and ancestral state reconstruction (see S5 Fig in Langille et al., [5]). Therefore, we chose to simply use each OTU representative sequence’s nearest-neighbor genome to infer genome contents. Specifically, to transform the OTU abundance table into a genome abundance table, the representative sequence of each OTU (query) is searched against a database composed of 16S rRNA sequences using USEARCH version 8.0.1623 with global alignment settings (-usearch_global) with fixed sequence identity cutoff (see below) specified in -id parameter. A genome that has the closest matched 16S rRNA sequence above the identity cutoff is considered as the inferred genome for that OTU. If there are more than one nearest-neighbor genomes with the same identities, the count is equally split among those genomes. The resulting genome abundance table is normalized by the 16S rRNA copy number of each genome. Then, genome content is inferred by the copy number of the genes within each inferred genome. Finally, inferred genome content of each genome bin is summed to generate total metagenomics content (KO abundances) of the sample. Content is expressed in terms of ortholog counts when using the KEGG genome database or RXN counts when using the BioCyc genome database (see S1 Text). Formally, if a sample contains *n* number of 16S rRNA OTUs (*t*), each representing nearest neighbor genome(s) *g* then the abundance of a single ortholog *K* in that sample is represented as *A*_*K*_ and can be calculated by:

AK=∑t=1nAt∑g=1mKcopygRcopygm

Where *A*_*t*_ is the abundance of OTU *t* in the sample. *Kcopy*_*g*_ is the copy number of *K* in the nearest neighbor genome and *Rcopy*_*g*_ is the copy number of the 16S rRNA genes in that same genome. Because an OTU can match equally well to more than one nearest neighbor genome, we represent the count of multiple matches as *m* and each is weighted equally.

### PICRUSt analyses

Raw 16S rRNA gene sequence data were fed to QIIME [22] (Amazon EC2 image AMI 1.9.1) and the PICRUSt pipeline to obtain functional count tables. First, paired-end sequencing reads were joined using join_paired_ends.py in QIIME. Then quality filtered and formatted by split_libraries_fastq.py in QIIME with default settings. Formatted sequences were clustered using pick_closed_reference_otus.py in QIIME with a similarity of 0.97. Then, the OTU table was fed to normalize_by_copy_number.py followed by predict_metagenomes.py with default settings to create the predicted metagenomics table.

### Tax4Fun analyses

QIIME was used to pre-process raw sequence data for Tax4Fun as described on the Tax4Fun website (http://tax4fun.gobics.de/). Formatted sequences were de novo clustered at 0.97 sequence similarity and representative sequences were selected. Then, taxonomic information was assigned using Silva 119 downloaded from the Tax4Fun website. An OTU table was created and fed to Tax4Fun R package. The Tax4Fun function was run with all default settings.

### Statistical tests

Statistical analyses were performed in the R environment (https://www.r-project.org/). The DESeq2 package was used to detect differentially abundant KEGG orthologs (KO) in cancer and healthy paired human oral biopsy samples for shotgun metagenomics, Piphillin and PICRUSt results. Fractional counts were floored to the nearest integer before analyses to allow count-based statistical procedures. Because Tax4Fun produces relative abundance estimates as opposed to counts, the Wilcoxon rank sum test was performed to detect differentially abundant features.

Differential abundance findings between inferred metagenomics and shotgun metagenomics were compared using the following parameters. True Positives, KOs passed q<0.2 in both inferred and shotgun metagenomics; True Negatives, all KOs in the database that did not pass q<0.2 in either inferred or shotgun metagenomics; False Positives, KOs passed q<0.2 in inferred metagenomics but not in shotgun metagenomics; False Negatives, KOs passed q<0.2 in shotgun metagenomics but not in inferred metagenomics.

## Results

### Identity cutoffs affect the quantity of retained sequences

**Fig 2:**
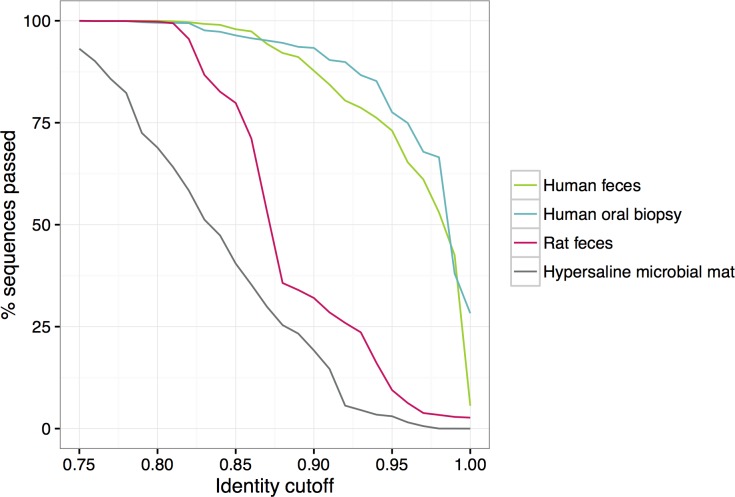
16S rRNA gene amplicon sequences passing the identity threshold to the reference genomes. Percentage of amplicon sequences from three datasets passing identity cutoffs from 0.75 to 1.00 against 16S rRNA gene sequences in the genome database were depicted. Green line, human feces dataset; blue line, human oral biopsy dataset; pink line, rat feces dataset; gray line, hypersaline microbial mat dataset.

In Piphillin, representative sequences of the OTUs and 16S rRNA gene sequences of genomes in the database are compared to infer genome contents (Fig 1). Thus, the identity threshold for considering a match affects results. An overly stringent sequence identity cutoff limits the number of 16S rRNA sequences used in the analysis, whereas a relaxed sequence identity cutoff adds noise and lowers the accuracy of inferred metagenomics. To examine the effect of sequence identity threshold, results using ten different cutoffs (0.75, 0.80, 0.85, 0.90, 0.95, 0.96, 0.97, 0.98, 0.99 and 1) were compared. As shown in Fig 2, the percentage of utilized total experimental sequencing data declines as the identity cutoff increases. The rate of decrease in the percentage of the sequence utilized was similar in human oral biopsy and feces, however, varied by other biospecimen type. Human oral biopsy samples maintained high sequence passed ratio of >90% up to 0.91 identity cutoff, whereas the hypersaline microbial mat sample dropped to <90% sequence passed ratio at 0.76 identity. None of the representative sequences from hypersaline microbial mat OTUs matched > = 99% to reference genomes and the percentage of passed sequences was the lowest among the three biospecimen types at all cutoffs, indicating relatively high abundance of unknown genomes in this biospecimen type. A similar trend was observed with BioCyc as a reference database (S1 Fig).

### Piphillin results correlate with shotgun metagenomics

**Fig 3:**
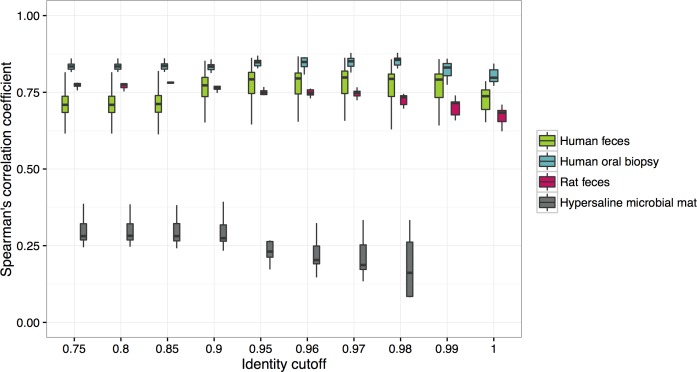
Spearman’s correlation coefficient between Piphillin results and shotgun metagenomics at ten different identity cutoffs tested in Piphillin. Spearman’s correlation coefficient was calculated for each sample and mean, 1^st^ and 3^rd^ quartiles are depicted by the boxes. Whiskers extend to the furthest points within 150% of the interquartile range. Green, human feces dataset; blue, human oral biopsy dataset; pink rat feces dataset; gray, hypersaline microbial mat dataset.

To examine how Piphillin results predict shotgun metagenomics, Spearman’s correlation coefficient was calculated between Piphillin results and corresponding shotgun metagenomics for each sample (Fig 3). Again, results varied depending on biospecimen types. Human oral biopsy, human feces and rat feces data sets exhibited relatively high correlation coefficients (0.61–0.88) compared to those of the hypersaline microbial mat data set (0.08–0.45) throughout the identity cutoffs tested (hypersaline microbial mat dataset limited to testing through 0.98 cutoff as discussed above). Correlation coefficients against shotgun metagenomics of human feces, human oral biopsy and rat feces were stable up to identity cutoff 0.99, 0.98 and 0.97, respectively, however, those of hypersaline microbial mat dropped at identity cutoff of 0.95. Using BioCyc as a reference database resulted in similar results with overall slightly higher correlation coefficients (S2 Fig).

### Functions with significantly different abundances are detected

**Fig 4:**
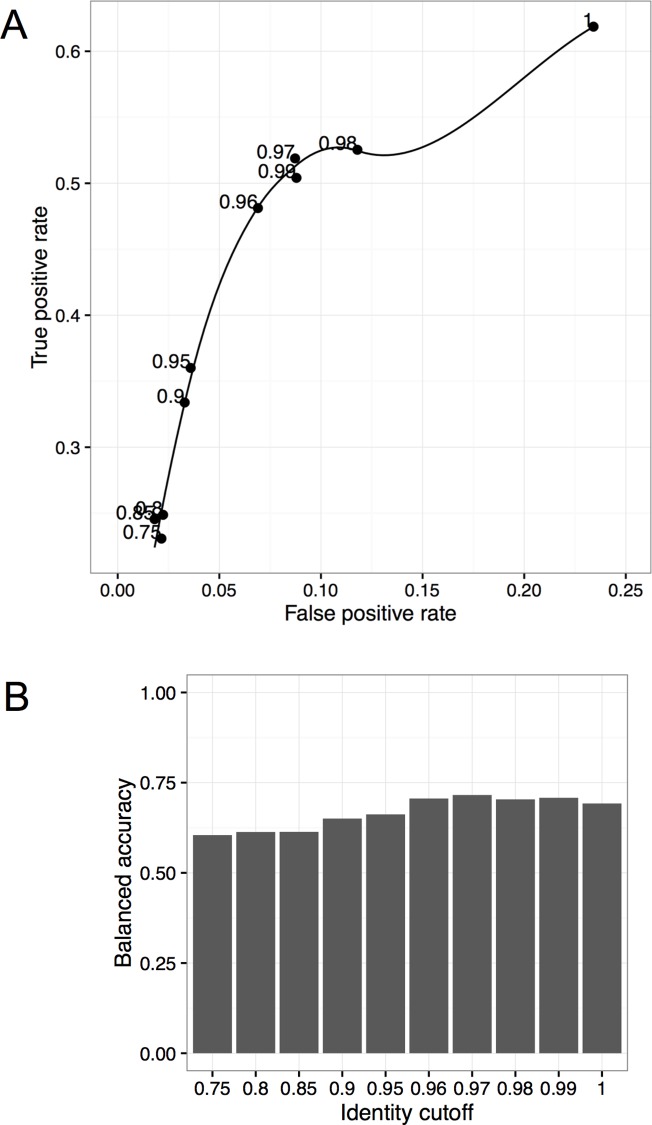
Sensitivity and specificity in identifying differentially abundant KOs from Piphillin against corresponding metagenomics. (A) True positive rate and false positive rate of detecting significantly differentially abundant KOs in human oral biopsy sample. Numbers next to each point represent identity cutoff used for Piphillin. (B) Balanced accuracy of Piphillin at each identity cutoff.

In many studies, identifying functional genes with significantly different abundances between treatment and control groups is one of the most important goals. Since we observed excellent correlations between Piphillin and shotgun metagenomics for clinical samples, we examined how well Piphillin can detect differentially abundant functions between cancerous or healthy oral biopsies with a paired negative binomial Wald test. For this validation, we applied the same test on shotgun metagenomics KO abundance tables of the corresponding samples. Piphillin results demonstrated that the number of differentially abundant KOs varied depending on the identity cutoff and they were slightly larger than those detected by shotgun metagenomics (Table 1). To examine sensitivity and specificity of the detection, the ROC curve was calculated by True Positive Rate (TPR; sensitivity) = True Positive / (True Positive + False Negative) and False Positive Rate (FPR; (1-specificity)) = False Positive / (False Positive + True Negative) (Fig 4A and S3A Fig). As identity cutoff increases, TPR increases along with increase of FPR. It should be noted that the range of FPR is 0.018–0.23 and they are 3–14 times smaller than the range of TPR 0.23–0.63. To identify the balance between TPR and FPR, we calculated balanced accuracy = TPR/2 + (1—FPR)/2 (Fig 4B and S3B Fig). With this biospecimen type, the peak balanced accuracy was observed at an identity cutoff of 0.97.

**Table 1:** Counts of KEGG Orthologs detected and differentially abundant as perceived by shotgun metagenomics and inferred metagenomics in human oral biopsy comparisons.

Data type	Nearest-neighbor genome identity cutoff	Count of KOs detected	Count of KOs detected per sample (max—min, median)	Count of differentially abundant KOs (q<0.2)
Metagenomics	NA	5,248	4,471–2,955, 3,817	611
Piphillin	0.75	6,818	6,391–4,456, 5,494	301
Piphillin	0.80	6,831	6,386–4,456, 5,473	318
Piphillin	0.85	6,759	6,372–4,451, 5,456	285
Piphillin	0.90	6,683	6,310–4,738, 5,374	449
Piphillin	0.95	6,506	6,008–4,039, 4,954	487
Piphillin	0.96	6,445	5,836–3,343, 4,694	807
Piphillin	0.97	6,431	5,813–3,379, 4,492	966
Piphillin	0.98	6,400	5,763–3,376, 4,456	1,198
Piphillin	0.99	6,196	5,564–2,851, 4,088	962
Piphillin	1	5,958	5,415–2,522, 3,858	2,119

### A contemporary genome database allows more accurate inference than the non-contemporary

To demonstrate the effect of database enrichment and importance of using up-to-date database, Piphillin results using KEGG release 73 (January 2015) was compared to those using KEGG release 71 (July 2014). KEGG release 71 was retrieved and formatted the same way as the release 73. During the short six month period, bacterial or archaeal genomes (defined by carrying 16S rRNA KO (K01977) with length of >1400bp and <1600bp) increased from 2,905 to 3,036 (131 additional genomes, about 5% increase). As a result, at identity cutoff of 0.97, Shannon diversity index was significantly increased (p<0.05, Wilcoxon rank sum test) and both TPR and FPR of the human oral biopsy study were increased 3% and 6%, respectively, when referencing the newer version of KEGG. This resulted in balanced accuracy increase from 0.714 to 0.719.

### Piphillin improves correlation and accuracy for the clinical samples

**Fig 5:**
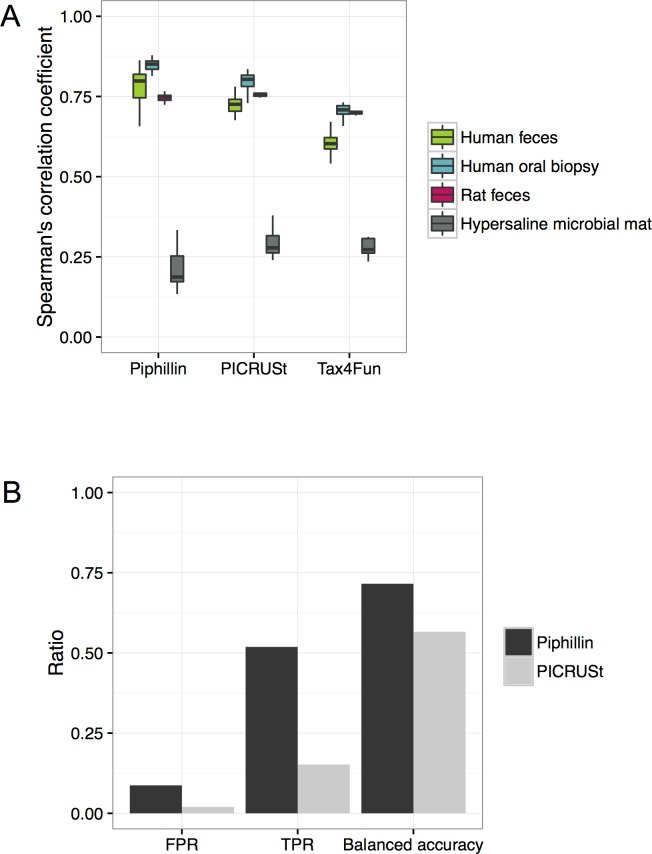
Comparison between Piphillin, PICRUSt and Tax4Fun. (A) Spearman’s correlation coefficient against corresponding shotgun metagenomics results were compared. Spearman’s correlation coefficient was calculated for each sample and ranges are depicted as box and whisker plots as described in Fig 3. Green, human feces dataset; blue, human oral biopsy dataset; pink rat feces dataset; gray, hypersaline microbial mat dataset. (B) False positive rate, true positive rate and balanced accuracy of detecting significant differences between cancer and healthy human oral biopsy samples were compared.

Of the currently available metagenomics inference tools, PICRUSt is the oldest and most cited and Tax4Fun is a recently published method that claimed improvements over PICRUSt. We compared performance of both tools to Piphillin. Since PICRUSt is wholly dependent on QIIME, QIIME was used to produce 16S rRNA OTU abundance table as described in the Methods section and then PICRUSt was run with all default settings. Since Tax4Fun is wholly dependent on the Silva ontology, Silva was used to process the sequencing reads as recommended by the Tax4Fun authors. The identity cutoff of 0.97 was used to produce Piphillin results. Total number of 16S rRNA sequences used in PICRUSt or Tax4Fun after QIIME pre-processing was 101% or 131%, respectively, of those used for Piphillin. First, Spearman’s correlation coefficient was compared between the three methods using the three datasets (Fig 5A). Piphillin produced significantly higher Spearman’s correlation coefficient compared to PICRUSt in the human feces and human oral biopsy dataset (Wilcoxon rank sum test, p<0.0001 and p<0.01, respectively) and Tax4Fun (Wilcoxon rank sum test, p<0.0001 and p<0.001, respectively), whereas the hypersaline microbial mat dataset demonstrated significantly lower Spearman’s correlation coefficient (Wilcoxon rank sum test, p<0.05 to both PICRUSt and Tax4Fun). There was no significant difference with the rat feces dataset to PICRUSt results (Wilcoxon rank sum test, p = 0.26) and significantly higher Spearman’s correlation coefficient to Tax4Fun results (Wilcoxon rank sum test, p<0.001). These results suggest that the capability of Piphillin, PICRUSt and Tax4Fun to predict shotgun metagenomic outcomes differs depending on biospecimen type, most likely due to how well the 16S rRNA gene sequences correspond to known genomes. Next, we compared FPR, TPR and balanced accuracy of human oral biopsy data set analyzed by different approaches (Fig 5B). Since Tax4Fun normalizes abundance of each KO to proportions, Wilcoxon rank sum test was used instead of DESeq2 to detect differentially abundant KOs. However, the test results did not produce any significant changes in KO abundances between healthy and cancer biopsies. This could be due to the sensitivity of the test to detect differential abundances in this cohort. Thus, we compared Piphillin and PICRUSt results against shotgun metagenomics results. Although FPR was three times higher with Piphillin compared to PICRUSt, TPR was also three times higher with Piphillin and balanced accuracy was 25% higher with Piphillin. In summary, Piphillin allows more false positives, however, overall accuracy is higher compared to PICRUSt in the human oral biopsy dataset.

## Discussion

We have developed Piphillin, a simple algorithm to predict metagenomes with high accuracy by leveraging the most-current genome reference databases. Piphillin has fewer parameter requirements and more flexibility compared to PICRUSt and Tax4Fun. With Piphillin, minimal formatting of reference genome databases is required because no genome segments need to be aligned or placed into a phylogenetic tree or clustered into reference OTUs. Further, unobserved ancestral genomes do not need to be imputed–avoiding highly parameterized (meaning changing a parameter leads to a different outcome) algorithms which are continually debated in the literature [23–30]. For example, some of the questions a biologist would need to answer and defend about his or her choice of methods would be: Which genes retain the evolutionary history? Which positions in these genes evolve independently? In which bacterial lineages have nucleotide transition and transversion probabilities been constant over time and which ones are time-nonhomogeneous?

Another advantage of Piphillin is its capability to receive data inputs from any upstream 16S rRNA amplicon sequence pre-processing pipeline since it is not restricted to QIIME’s nor Silva’s assignments of counts (Table 2). It can also be used in conjunction with Mothur [31], RDP [32], DADA2 [33] or UPARSE [34]. Piphillin requires only two input files from the user, an OTU abundance table and a fasta file with OTU representative sequences. Considering the increasing rate of publication of sequenced bacterial and archaeal genomes and the observed benefit of incorporating all available reference genomes, choosing the tool that can adapt rapidly to new knowledge is crucial for increasing the accuracy of metagenomic inference. The fundamental characteristics that allow Piphillin to adapt rapidly include obviating the dependencies upon 1) a multiple sequence alignment of all phylogenetically informative 16S rRNA genes (over 1.8 million genes in the NCBI Nucleotide database), 2) phylogenetic tree calculation and bootstrapping, 3) creation of reference OTUs, and 4) assignment of new identifiers for novel branches within QIIME or Silva. By contrast, PICRUSt requires each of these steps, which are not just computationally time-consuming but employ methods that are open to argument, as discussed above. These facts make it difficult to create a stable tree and update it constantly. In fact, the current reference tree supported by PICRUSt (Greengenes version 13_5) is already three years old due to many of the reasons above. By contrast, Piphillin requires the unaligned 16S rRNA gene sequences of each genome and the corresponding genome contents as the reference database. Hence, the Piphillin database can easily be updated with new genome sequences, improving the prediction accuracy. Genome inference of Piphillin relies on direct nearest-neighbor 16S rRNA gene sequences in a given genome database. This design allows more flexibility in choosing and updating a genome database and allows even novice users to easily comprehend the single parameter chosen at runtime, the identity cutoff. We have examined ten different identity cutoffs ranging from 0.75 to 1 by correlation of the results to the observed shotgun metagenomes as well as detection of KOs with significant abundance differences. Correlations between Piphillin and shotgun metagenomics varied depending on the biospecimen type (Fig 3), as did the proportions of 16S rRNA reads matching reference genomes (Fig 2). Sequences that do not pass the identity threshold to 16S rRNA sequences in the database are not used in Piphillin. Thus, the more sequences in the sample that are dissimilar to the reference database, the lower the correlation to the shotgun metagenomics. The Piphillin approach is most suitable for biospecimens with a high content of resident organisms with sequenced genomes, such as human clinical biospecimens.

**Table 2:** Piphillin has less pre-requisites than PICRUSt and Tax4Fun.

		Piphillin	PICRUSt	Tax4Fun
Database pre-requisites				
	16S rRNA gene sequences of each genome	✔	✔	✔
	Multiple sequence alignment		✔	✔
	Phylogenetic tree building		✔	✔
	Inference of gene contents in ancestral genomes		✔	
Input data pre-requisites				
	OTU abundance table	✔	✔	✔
	OTU representative sequence	✔	✔	✔
Data processing pre-requisites				
	QIIME		✔	
	SilvaNGS or QIIME+Silva extension or Silva identifiers)			✔
	UProC or PAUDA annotation of each genome			✔
	Closed OTU picking with reference tree leaves		✔	✔

**Fig 6:**
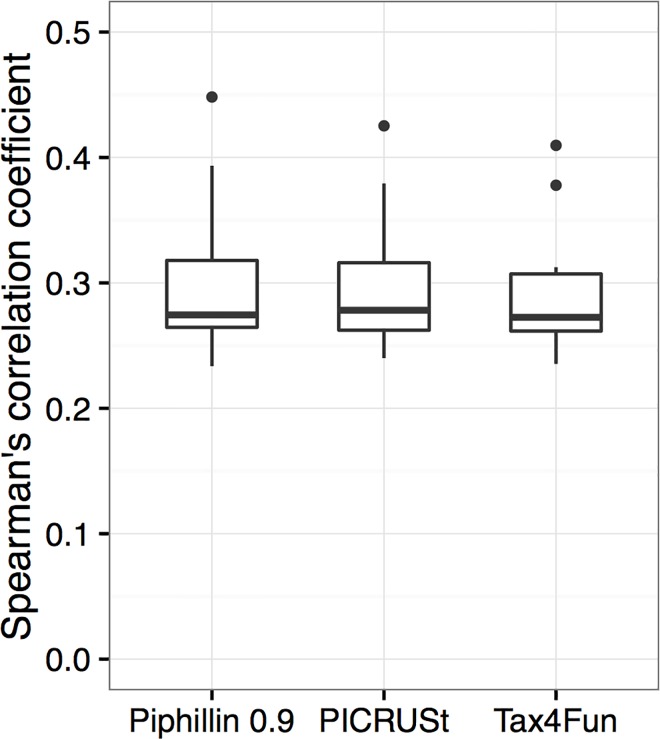
Comparison between Piphillin with 0.9 identity cutoff and two other approaches. Spearman’s correlation coefficient against shotgun metagenomics results was calculated for hypersaline microbial mat dataset. Ranges are depicted as box and whisker plots as described in Fig 3.

We compared metagenomics inference performance of Piphillin to PICRUSt and Tax4Fun. Correlation with shotgun metagenomics was significantly improved by Piphillin compared to the other two approaches for human feces and human oral biopsy datasets. However, PICRUSt and Tax4Fun produced higher correlations for hypersaline microbial mats (Fig 5A). This result is expected due to the large proportion (99.4%) of sequences in the hypersaline microbial mat not passing the 97% identity cutoff (Fig 2). In such cases, identification of nearest-neighbor genomes is improved by using a lower identity cutoff. Indeed, at a 90% identity cutoff, there were no significant differences between Spearman’s correlation coefficients between Piphillin and PICRUSt or Tax4Fun (Fig 6; Wilcoxon rank sum test p = 0.97 or 0.85, respectively). Overall, all three methods performed poorly in predicting the shotgun metagenomes from the environmental sample (Spearman’s correlation coefficient, 0.23–0.45) indicating the need for intensive genome sequencing of diverse microbes from environmental sources before metagenomics inference can become reliable for environmental microbiome science.

FPR and TPR of significantly changed KOs in Piphillin against shotgun metagenomics according to DESeq2 analysis were examined using a human oral biopsy dataset (Fig 4A). The identity cutoff of 0.97 produced the highest balanced accuracy. However, the difference of balanced accuracy between a cutoff of 0.97 identity and cutoffs between 0.96 and 1 are about 1% (Fig 4B). This result suggests that any identity cutoff between 0.96 and 1 results in approximately equivalent balanced accuracy. Our analyses suggested that in the human oral biopsy sample, a lower identity cutoff diminishes TPR, however, it also maintains low FPR. Thus, depending on the downstream application, a researcher can focus on identifying a smaller list of truly differentially abundant KOs and accept a diminished sensitivity by selecting a low identity cutoff. The lower identity cutoff also allows utilization of a greater number of amplicon sequences from the biospecimens.

Surprisingly, the human fecal dataset pre-processing for Tax4Fun took ten days on a 32 CPU server with 60 Gb RAM following the Tax4Fun recommended parallel_assign_taxonomy_blast.py alignment of representative sequence from each OTU against the Silva database for taxonomical assignment. Since the analogous step in Piphillin completes in less than one minute on a single CPU with 4Gb RAM, we expect that Tax4Fun will experience speed improvements once tuned for large scale clinical datasets.

It should be noted that our choice of DESeq2 for identifying KOs with differential abundance (feature selection) in both shotgun metagenomics and inferred metagenomics directly from count data is based on substantial improvements in microbiome analysis over rarefying counts or standardizing to proportions [35]. DESeq2 has been shown to be appropriate for feature selection when 1) the dispersion of observed counts of a minority feature across samples is greater than the dispersion for frequently encountered features [S2 Text, S4A–S4C Fig], 2) the depth of sequencing is inconsistent among samples, 3) certain features in the abundance table can be omitted before multiple testing adjustment by independent filtering, and 4) counts fit a negative binomial distribution [36]. The first three conditions are met. The fourth condition is difficult to justify. In inferred functional gene counting, one 16S rRNA read infers many increments of functional gene counts and sometimes these increments are fractions in the case of a 16S rRNA gene equally matching multiple genomes and also when a genome’s 16S rRNA copy number and its copy number for a particular KO is not equal. These non-discrete increments are expected to cause violations in fitting the negative binomial distribution, condition 4, above. Popular software such as TopHat’s fusion transcript mapper [37] avoid fractional increments by dismissing cases where a read matches equally-well to multiple references but in the broad search space necessary for metagenomics applications, ignoring these reads would result in biased observations. Therefore, we decided to retain the split counts and simply determine if the DESeq2 feature selection from Piphillin and PICRUSt predicted the DESeq2 results from shotgun metagenomics. The results showed both Piphillin and PICRUSt outputs were able to predict the majority of differences observed in shotgun metagenomics but Piphillin had greater accuracy. A non-parametric test such as Wilcoxon rank sum test may be an alternative to DESeq2 for searching for experimental associations in these outputs. However, the Wilcoxon rank sum test is less sensitive to detecting differential abundance especially in small numbers of samples [38] and in fact, no significantly different KO was detected in shotgun metagenomics, Piphillin and PICRUSt results.

The limitation of Piphillin is the lack of nearest-neighbor reference genomes in understudied environments. However, recent efforts in sequencing genomes by HMP (http://www.commonfund.nih.gov/hmp), MetaHIT consortium (http://www.metahit.eu/) and genome assemblies from complex samples [39] are expanding our knowledge of bacterial genomes rapidly, especially in clinical samples. With the Piphillin algorithm, those sequenced genomes are added easily to the reference database, which leads to increased accuracy of metagenomics inference. Piphillin’s rapid inclusion of new genome sequences will contribute to the detection and better understanding of functional changes in previously published and future studies.

## Supporting Information

S1 Fig 16S rRNA gene amplicon sequences passing the identity threshold to the reference BioCyc genomes. Percentage of amplicon sequences from three datasets passing identity cutoffs from 0.75 to 1.00 to 16S rRNA gene sequences in BioCyc genome database were depicted. Solid line, human oral biopsy dataset; dotted line, rat feces dataset; dashed line, hypersaline microbial mat dataset.
(TIFF)

S2 Fig Spearman’s correlation coefficient between BioCyc Piphillin results and shotgun metagenomics at ten different identity cutoff tested in Piphillin. Spearman’s correlation coefficient was calculated for each sample and mean, 1^st^ and 3^rd^ quartiles are depicted by the boxes. Whiskers extend to the furthest points within 150% of the interquartile range. Green, human oral biopsy dataset; blue rat feces dataset; pink, hypersaline microbial mat dataset.
(TIFF)

S3 Fig Sensitivity and specificity in identifying differentially abundant BioCyc RXNs from Piphillin against corresponding shotgun metagenomics. (A) True positive rate and false positive rate of detecting significantly differentially abundant BioCyc RXNs in human oral biopsy sample. Numbers next to each point represents identity cutoff used for Piphillin. (B) Balanced accuracy of BioCyc Piphillin at each identity cutoff.
(TIFF)

S4 Fig Dispersion plot showing the dispersion against the mean of normalized counts of human oral biopsy samples. (A) Metagenomics distribution. (B) KEGG Piphillin distribution. (C) PICRUSt distribution.
(TIFF)

S1 Text BioCyc as a reference database for Piphillin. (DOCX)

S2 Text DESeq2 to test differential abundance from Piphillin results. (DOCX)
