# Supplementary figures and images for: Piphillin: Improved Prediction of Metagenomic Content by Direct Inference from Human Microbiomes
Source: PLoS One. 2016 Nov 7;11(11):e0166104. doi: 10.1371/journal.pone.0166104 (PMC5098786; doi:10.1371/journal.pone.0166104)

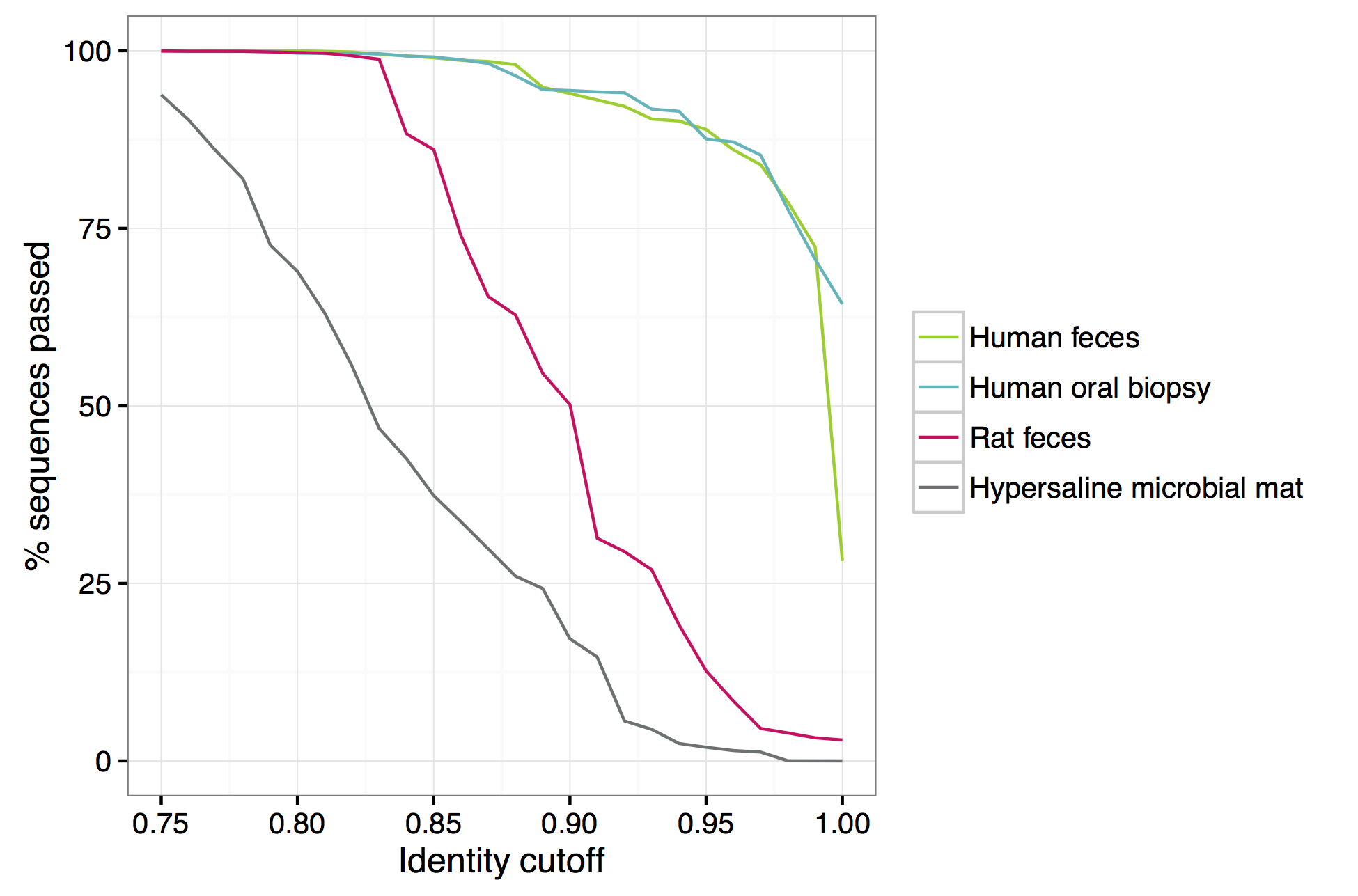

Supplement: S1 Fig — Percentage of amplicon sequences from three datasets passing identity cutoffs from 0.75 to 1.00 to 16S rRNA gene sequences in BioCyc genome database were depicted. Solid line, human oral biopsy dataset; dotted line, rat feces dataset; dashed line, hypersaline microbial mat dataset. (TIFF) [file pone.0166104.s001.tiff]

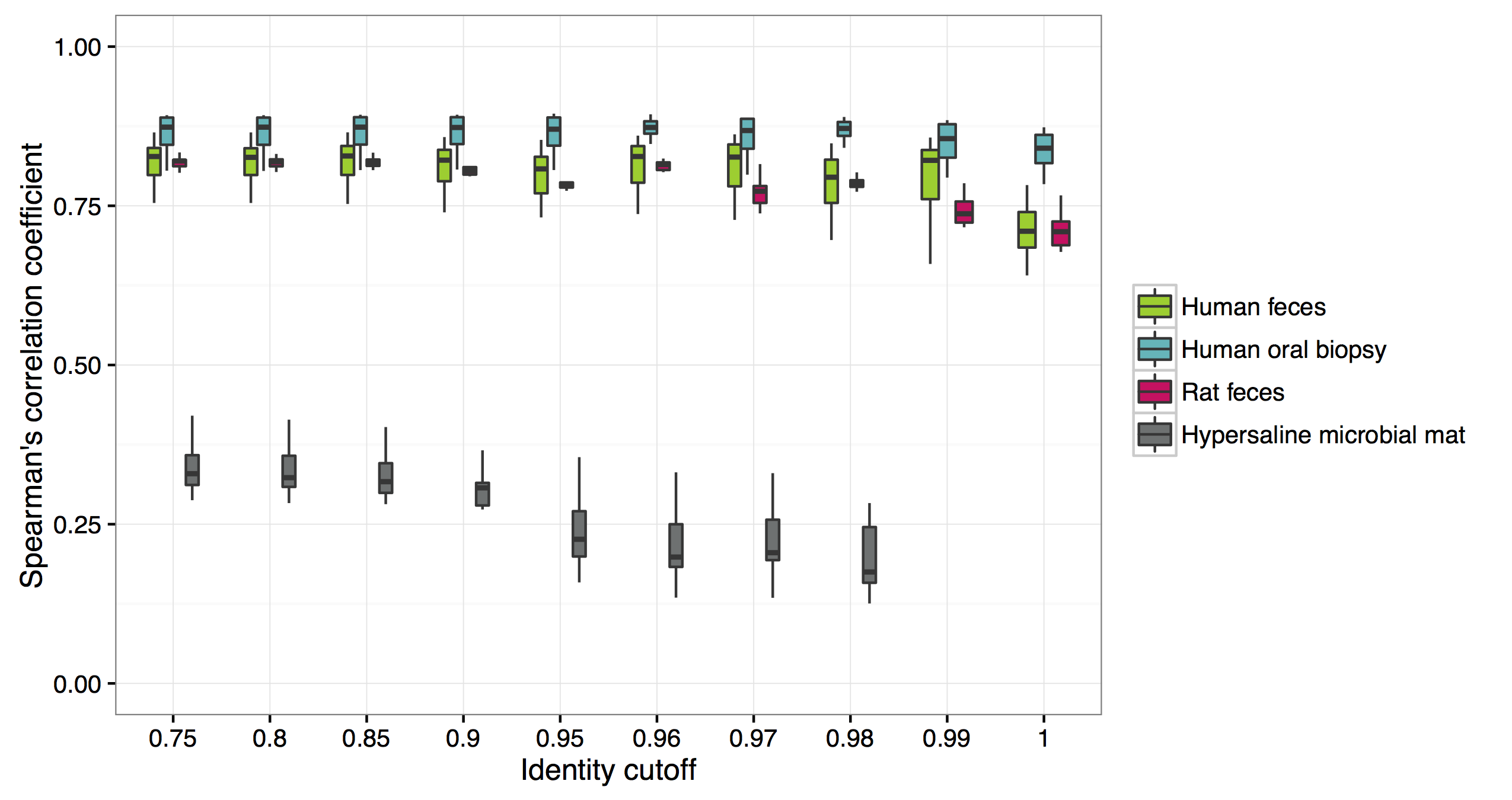

Supplement: S2 Fig — Spearman’s correlation coefficient was calculated for each sample and mean, 1st and 3rd quartiles are depicted by the boxes. Whiskers extend to the furthest points within 150% of the interquartile range. Green, human oral biopsy dataset; blue rat feces dataset; pink, hypersaline microbial mat dataset. (TIFF) [file pone.0166104.s002.tiff]

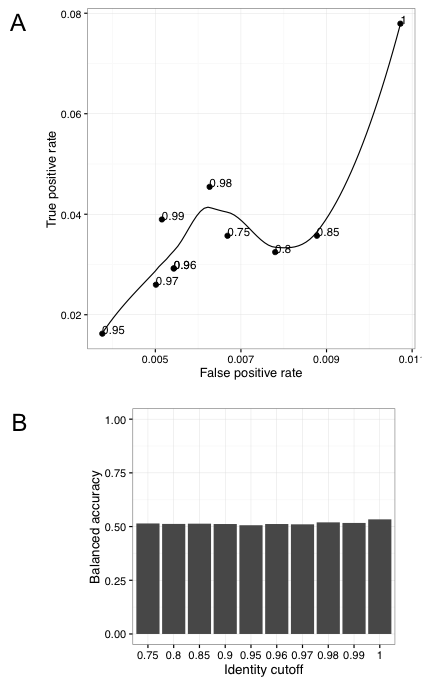

Supplement: S3 Fig — (A) True positive rate and false positive rate of detecting significantly differentially abundant BioCyc RXNs in human oral biopsy sample. Numbers next to each point represents identity cutoff used for Piphillin. (B) Balanced accuracy of BioCyc Piphillin at each identity cutoff. (TIFF) [file pone.0166104.s003.tiff]

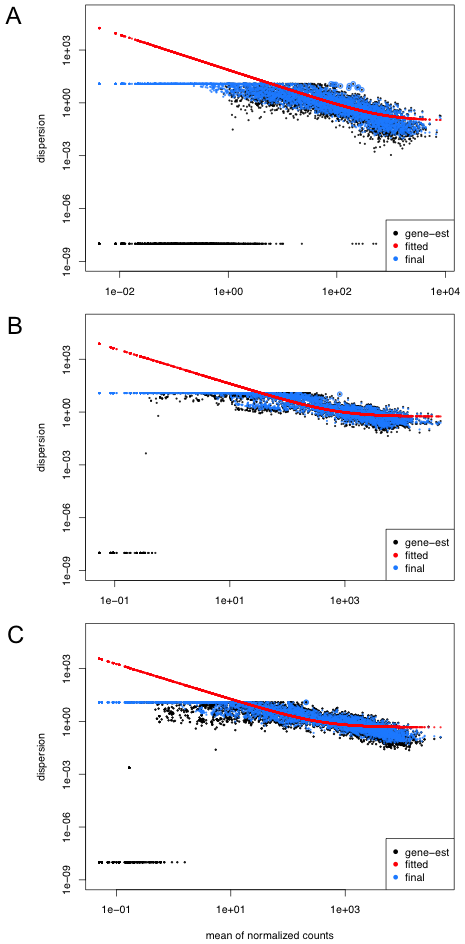

Supplement: S4 Fig — (A) Metagenomics distribution. (B) KEGG Piphillin distribution. (C) PICRUSt distribution. (TIFF) [file pone.0166104.s004.tiff]
