# Supplementary material for: Piphillin: Improved Prediction of Metagenomic Content by Direct Inference from Human Microbiomes
Source: PLoS One. 2016 Nov 7;11(11):e0166104. doi: 10.1371/journal.pone.0166104 (PMC5098786; doi:10.1371/journal.pone.0166104)
Supplement: S1 Text — (DOCX) [file pone.0166104.s005.docx]

**S1 Text. BioCyc as a reference database for Piphillin**

**Methods**

Gene copy numbers were retrieved from BioCyc 18.5 to create a gene feature table. From each genome sequence, 16S rRNA gene IDs were extracted using keyword “16S ribosomal RNA” as a “COMMON-NAME” in rnas.dat file in each BioCyc PGDB. Corresponding fasta format 16S rRNA sequences were retrieved and filtered using length cutoff of >1400bp and <1600bp. Number of 16S rRNA gene sequences passing the length filter in each genome was used to normalize 16S rRNA copy numbers. Gene copy numbers for each genome were retrieved, summarized by BioCyc reactions (RXNs) and formatted using a custom script for the database.

**Results and Discussion**

**Identity cutoffs and passed sequences using BioCyc database**

Sequence identity cutoffs to infer BioCyc genomes for each OTU were compared using the same method described in the main text. As shown in S1 Fig, similar to KEGG, the percentage of total experimental sequencing data utilized declines as identity cutoff increases. The percentages of utilized OTUs are higher in many cases in BioCyc compared to KEGG at the same identity cutoffs. Sequence passed ratio of >90% was 0.94, 0.94, 0.83, and 0.76 for human feces, human oral biopsy, rat feces and hypersaline microbial mat datasets, respectively, with BioCyc, compared to 0.88, 0.91, 0.82 and 0.76 for the same datasets with KEGG. This observation is probably due to a larger number of microbial 16S rRNA genes (defined by annotated 16S rRNA gene sequence length >1400bp and <1600bp) in BioCyc (4,392 genomes) compared to KEGG (3,037 genomes).

**Correlation between BioCyc Piphillin results and shotgun metagenomics**

Shotgun metagenomic sequences were searched against BioCyc genes using RAPSearch with the same settings described in the main text and counts were summarized by BioCyc RXNs to compare with Piphillin results. A slightly better correlation of BioCyc Piphillin to metagenome results than KEGG Piphillin (S2 Fig) was observed, which is probably due to the denser reference database and use of more sequences in the Piphillin output. The human oral biopsy dataset demonstrated a median correlation coefficient of 0.84 even at identity cutoff of 1. By contrast, the correlation coefficient of the hypersaline microbial mat dropped at an identity cutoff of 0.95, as also observed in KEGG.

**Detection of significantly different functions by BioCyc Piphillin**

Despite higher correlations between BioCyc Piphillin and shotgun metagenomics, TPR was an order of magnitude lower in BioCyc Piphillin compared to KEGG Piphillin, which was also true for FPR and Balanced Accuracy (S3A + S3B Fig). This observation might be attributed to the bin size differences between KEGG KOs and BioCyc RXNs. KEGG KOs are groups of orthologous genes whereas BioCyc RXNs are groups of genes that contribute to a single step of an enzymatic reaction or a transport between cellular compartments. In the BioCyc collection of databases, genes that perform the same RXN have the same identifier in different genomes. When different identifiers were encountered for genes performing the same RXN, we used the identifier contained in MetaCyc whenever possible. As Piphillin is applied to biospecimens of diverse origins, we may continue to find that the KEGG KO ontology is better suited for predicting differential abundances.
