# Supplementary material for: Piphillin: Improved Prediction of Metagenomic Content by Direct Inference from Human Microbiomes
Source: PLoS One. 2016 Nov 7;11(11):e0166104. doi: 10.1371/journal.pone.0166104 (PMC5098786; doi:10.1371/journal.pone.0166104)
Supplement: S2 Text — (DOCX) [file pone.0166104.s006.docx]

**S2 Text. DESeq2 to test differential abundance from Piphillin results**

DESeq2 was originally developed for RNA-seq count data and expects a negative binomial distribution of the counts to estimate dispersions. Since Piphillin and PICRUSt involve multiple steps to estimate functional counts from original 16S rRNA sequencing counts, we evaluated whether the statistical testing using DESeq2 is appropriate for Piphillin or PICRUSt results. Estimated dispersions were plotted against mean of normalized counts (S4 A-C Fig). Gene-wise estimate was slightly wider in shotgun metagenomics (S4A Fig) compared to Piphillin (S4B Fig) or PICRUSt (S4C Fig), while the fitted curve seemed to be similar among all three cases.
